# Analysis of Singlet Oxygen Luminescence Generated By Protoporphyrin IX

**DOI:** 10.3390/antiox14020176

**Published:** 2025-01-31

**Authors:** Vikas Vikas, Weibing Yang, Brian C. Wilson, Timothy C. Zhu, Robert H. Hadfield

**Affiliations:** 1 Electronic & Nanoscale Engineering, James Watt School of Engineering, University of Glasgow, Glasgow G128QQ, UK; 2 Department of Radiation Oncology, University of Pennsylvania, Philadelphia, PA 19104, USA; weibing.yang@pennmedicine.upenn.edu (W.Y.); timothy.zhu@pennmedicine.upenn.edu (T.C.Z.); 3 Department of Medical Biophysics, University Health Network/University of Toronto, Toronto, ON M5G 2C4, Canada; brian.wilson@uhn.ca

**Keywords:** reactive oxygen species, singlet oxygen, photodynamic therapy, protoporphyrin IX, time-resolved singlet oxygen luminescence detection

## Abstract

The effectiveness of photodynamic therapy (PDT) for cancer treatment relies on the generation of cytotoxic singlet oxygen (^1^O_2_) in type II PDT. Hence, monitoring of ^1^O_2_ generation during PDT enables optimal treatment delivery to the tumor target with reduced off-target effects. Direct ^1^O_2_ observation by measuring its luminescence at 1270 nm remains challenging due to the very weak signal. This study presents ^1^O_2_ luminescence measurements using a time-resolved singlet oxygen luminescence detection system (TSOLD) applied to protoporphyrin IX (PpIX) in different solvents (ethanol and acetone) and biological media (bovine serum albumin and agarose-based solid phantom). The compact experimental setup includes a nanosecond diode laser with a function generator, a cuvette with photosensitizer solution, optical filtering and mirrors, an InGaAs single-photon avalanche diode detector, and time-tagger electronics. Increasing the concentration of PpIX in these media from 1 to 10 µg/g resulted in a 3–5 × increase in the ^1^O_2_ luminescence signal. Furthermore, increasing light scattering in the sample using Intralipid from 0.1 to 1% led to a decrease in the ^1^O_2_ luminescence signal and lifetime. These results confirm the marked effect of the microenvironment on the ^1^O_2_ signal and, hence, on the photodynamic efficacy.

## 1. Introduction

**Figure 1:**
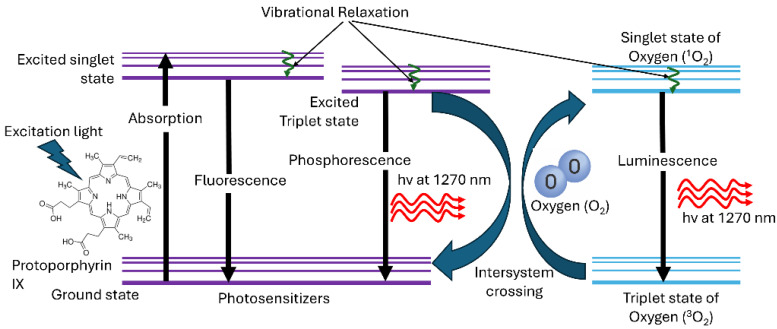
Jablonski diagram illustrating the generation of the 1270 nm near-infrared luminescence emission of singlet oxygen (^1^O_2_) and triplet-state photosensitizer luminescence.

Photodynamic therapy (PDT) is a minimally invasive therapeutic modality that has been used for a variety of malignant and non-malignant conditions [1,2,3]. PDT for cancer treatment utilizes light, a photosensitizer (PS) and molecular oxygen to induce localized cell death by multiple mechanisms such as cell death by immune response [3,4,5]. In the so-called Type I pathway, the photoexcited triplet-state photosensitizer interacts with cellular substrates, most commonly membranes, to produce free radicals and reactive oxygen species (ROS) such as superoxide, hydroxyl radicals and hydrogen peroxide [6,7]. As shown in Figure 1, in the Type II pathway that pertains to most clinical photosensitizers, the excited triplet-state PS transfers energy to triplet ground-state molecular oxygen (^3^O_2_), generating highly reactive singlet oxygen (^1^O_2_) [8,9,10]. Hence, sensitive monitoring of ^1^O_2_ during PDT should enable optimal treatment delivery to the tumor target with reduced off-target effects [3]. A variety of approaches have been investigated for PDT dosimetry, including biophysical/biological tissue response monitoring, explicit dosimetry in which the light, photosensitizer and tissue oxygenation are measured and combined into a biophysical model, and implicit dosimetry in which a surrogate measure such as photosensitizer photobleaching is used [4,10,11].

The direct technique of singlet oxygen luminescence dosimetry (SOLD) uses observation of the 1270 nm near-infrared luminescence emission of the ^1^O_2_ → ^3^O_2_ transition [12,13,14]. Since this directly quantifies the concentration of cytotoxic species, it can be considered the “gold standard” for PDT dosimetry. However, measurement is challenging due to the very low signal and the short half-life in biological media. Detection is significantly improved by the use of single-photon counting using NIR photomultiplier tubes (PMTs), InGaAs single-photon avalanche diodes (SPADs) and superconducting nanowire single-photon detectors (SNSPDs) [15,16,17,18]. Time-resolved single-photon counting (TSOLD) and gating allow for the subtraction of the contribution of the substantial background due to photosensitizer fluorescence and phosphorescence [13,17,19,20,21], while fitting the temporal spectrum yields the singlet oxygen and PS triplet-state lifetimes. Typically, measurements are made on and at wavelengths either side of the 1270 nm peak to subtract the residual background after long-pass filtering of the total emission. TSOLD has been validated through multiple in vitro and in vivo studies in cells, tumors and normal tissues [13,22].

TSOLD can be implemented using commercially available SPADs and SNSPDs with low dark count rates, low timing jitter and free-running operation. Here, we developed a versatile TSOLD testbed that incorporates a nanosecond diode excitation laser with a function generator that provides external triggering to select a suitable repetition rate, a cuvette containing the photosensitizer solution, custom optical filtering and mirrors, an InGaAs SPAD detector and time-resolved electronics.

The choice of photosensitizer plays a critical role in the efficacy and specificity of PDT treatments. An ideal photosensitizer should possess a high ^1^O_2_ quantum yield, strong absorption in the therapeutic window (~600–800 nm) and selective accumulation in the target cells/tissues [2,23]. Porphyrin-based compounds, such as porfimer sodium (Photofrin^®^), and protoporphyrin IX (PpIX) synthesized in target cells by administration of aminolevulinic acid, were among the first photosensitizers approved for clinical use [24,25]. However, there is a gap in knowledge of the lifetime and efficacy of ^1^O_2_ produced by PpIX photoactivation in various microenvironments [26]. Here, we performed a comprehensive study utilizing our in-house TSOLD system with PpIX in ethanol, acetone, bovine serum albumin (BSA) and agarose-based solid phantoms. The impact of light scattering on the ^1^O_2_ generation was determined by adding the lipoprotein Intralipid with either ethanol or methanol solvents to mimic a tissue-like microenvironment. This study of ^1^O_2_ luminescence detection by PpIX in various solvents and biological media is the first set towards future applications such as dosimetry for PDT.

## 2. Materials and Methods

### 2.1. Time-Resolved ^1^O_2_ Luminescence System

**Figure 2:**
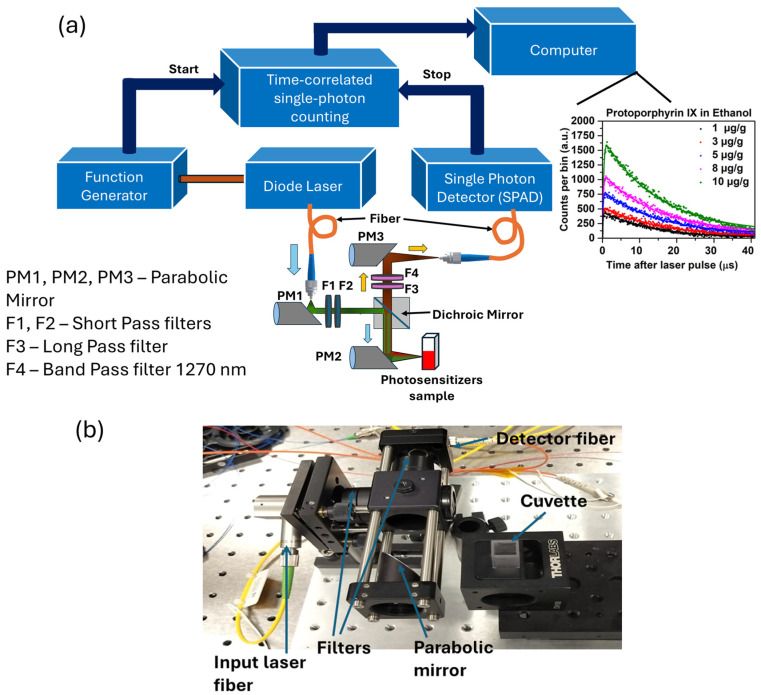
(**a**) Schematic and optical setup of the TSOLD experiment (upper). An optical beam from a nanosecond diode laser with a function generator illuminates the photosensitizer cuvette through a parabolic mirror and short-pass filters. The collected 1270 nm luminescence emission is coupled into an InGaAs-SPAD. TCSPC then generates time histograms of the ^1^O_2_ luminescence signal. (**b**) A photograph of the optical setup is shown (lower).

The system (Figure 2) is designed to measure the near-infrared singlet oxygen luminescence that comprises a nanosecond diode excitation laser with a function generator, a quartz cuvette (CV10Q35, Thorlabs, Newton, NJ, USA) containing photosensitizer solution, customized optical filtering and mirrors, a single-photon avalanche diode (SPAD) detector, and time-tagger electronics for time-correlated single-photon counting (TCSPC).

The 6–129 ns pulse-width tunable diode laser (NPL52C: Thorlabs, Newton, NJ, USA) has a broad spectral output of 515–525 nm and a variable repetition rate up to 50 kHz. The wavelength of the illuminated laser (520 nm) was selected corresponding to the secondary absorption region of PpIX. The output is coupled into a multimode optical fiber (core diameter 400 µm, 0.22 numerical aperture (M146L01, Thorlabs, Newton, NJ, USA)) connected to a collimation system consisting of a parabolic mirror (RC04FC-P01, Thorlabs, Newton, NJ, USA) and 25 mm diameter short-pass (optical density OD = 5.0 at >950 nm) (FESH0950, Thorlabs, Newton, NJ, USA) and band-pass (304–785 nm: out-of-band OD = 4.0) (FGS550, Thorlabs, Newton, NJ, USA) filters to produce a 4 mm collimated beam that is reflected by a dichroic mirror (DMLP950, Thorlabs, Newton, NJ, USA) and focused into the cuvette using a parabolic mirror to illuminate the sample. The 1270 nm luminescence emission passes through a dichroic mirror and the combination of long-pass (OD = 5.0 at <1200 nm: FELH1200, Thorlabs) and band-pass filters (bandwidth 1260–1280 nm, out-of-band OD = 6.0) (1270BP20, Omega Optical, Brattleboro, VT, USA). The beam is then coupled to a multimodal, 65 μm core diameter fiber to the SPAD detector (ID230, IDQ, Geneva, Switzerland) using a parabolic mirror. The TCSPC module (Time Tagger Ultra, Swabian Instruments, Stuttgart, Germany) counts the input signals from the function generator, sending a START signal through an electrical synchronization pulse, and the detector output then provides the STOP signal. Temporal histograms are thereby generated from single-photon detection events. Pulse pile-up can cause errors in the determination of the ^1^O_2_ lifetime so that the detection count rate is kept below 5% of the excitation laser pulse rate.

### 2.2. Photosensitizer in Various Media

**Figure 3:**
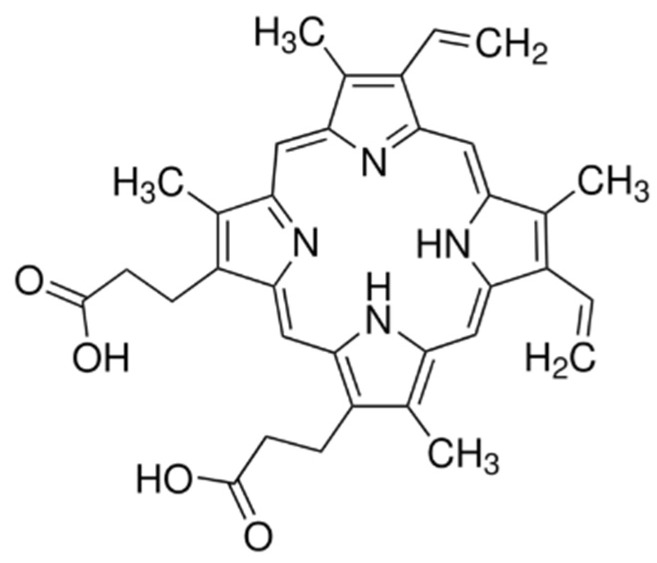
Molecular structure of PpIX [28].

The photosensitizer solutions were prepared by dissolving protoporphyrin IX (PpIX) (P8293, Sigma Aldrich, St. Louis, MO, USA) powder in ethanol or acetone. The molecular weight of PpIX is 562.66 g/mol and its molecular structure is shown in Figure 3. For the bovine serum albumin (BSA) and solid phantoms, the PpIX was dissolved first in dimethyl sulfoxide (DMSO). A 50 µg/g BSA solution was prepared by dissolving BSA powder (A9647, Sigma Aldrich) in phosphate-buffered saline (PBS). The solid phantoms were prepared using 1% agarose (A9539, Sigma Aldrich, St. Louis, MO, USA) dissolved in deionized water as the base material [27], mixed with the PpIX-DMSO solution and maintained at room temperature (20–25 °C) to solidify. For each TSOLD experiment, the PpIX was used at 1, 3, 5, 8 and 10 µg/g concentrations in 1 mL samples.

### 2.3. Determination of Singlet Oxygen Lifetime

Singlet oxygen generation occurs due to energy transfer from a triplet-state photosensitizer exchanging energy with triplet ground-state oxygen (^3^O_2_)_._ For short pulse activation (≤100 ns), the lifetime of singlet oxygen and triplet-state photosensitizers at time *t* following the pulse is given by Refs. [12,29]:

(1) $$\left[1_{O_{2}}\right]\left(t\right)=N\sigma \left[S_{0}\right]\varphi_{D}.\;\frac{\tau_{D}}{\tau_{T}-\tau_{D}}\left(\exp\left(\frac{-t}{\tau_{T}}\right)-\exp\left(\frac{-t}{\tau_{D}}\right)\right)$$

where [^1^O_2_] (*t*) is the singlet oxygen concentration, *N* is the number of photons per pulse, *σ* is the photosensitizer absorption cross-section at the excitation wavelength, [*S*_0_] is the photosensitizer concentration, *φ_D_* is the photosensitizer singlet oxygen quantum yield, and $\tau_{D}$ and $\tau_{T}$ are the lifetimes of the singlet oxygen and triplet-state photosensitizer, respectively. After subtracting the background signal due to other sources of near-infrared emission, the measured time histograms were fitted using Equation (1) to calculate the two lifetimes.

### 2.4. Validation of TSOLD System

Before measuring the ^1^O_2_ luminescence signature from PpIX in different microenvironments, the TSOLD system performance was validated against measurements made with a set of 20 nm bandwidth band-pass filters centered at 1200, 1240, 1270, 1300 and 1340 nm. A solution of 10 µg/g PpIX in ethanol was illuminated by the 520 nm laser beam (520 nm ± 7.5 nm, 129 ns at 25 kHz) with a beam waist of 0.2 mm and an average intensity of 32 mW.mm^−2^. Signal acquisition was conducted with the SPAD, operating with 10% single-photon detection efficiency and a 41 µs dead time.

### 2.5. ^1^O_2_ Luminescence from PpIX in Ethanol and Acetone

Measurements were made using 10 min signal integration with 1–10 µg/g PpIX solution in 1 mL cuvettes with a path length of 10 mm, using a 1 mm diameter beam at 520 nm ± 7.5 nm, at 20 or 3.2 mW.mm^−2^ in ethanol and acetone, respectively. The SPAD was operated at 10% efficiency with 41 µs dead time to avoid pulse pile-up. Histograms were generated with 610 bins with a 65 ns width. These were fitted using Equation 1 using the Levenberg–Marquardt algorithm.

### 2.6. ^1^O_2_ Luminescence from PpIX in Biological Media

Measurement of singlet oxygen generation in cells and tissues is of value for dosimetry in photodynamic therapy. BSA and tissue-mimicking phantoms were excited by a 2 mm diameter laser beam at 5.5 mW.mm^−2^, 129 ns pulse width and 30 kHz repetition rate. The luminescence was measured after passing through a 1270 nm, 20 nm bandwidth filter using a SPAD with 25% efficiency and 34 µs dead time. Time histograms of 520 bins with a bin width of 65 ns were generated by capturing single-photon arrival events using the Time Tagger. These plots were fitted with Equation 1, used to calculate the ^1^O_2_ and photosensitizer triplet-state lifetimes.

### 2.7. Impact of Scattering on Singlet Oxygen Generation and Luminescence Lifetime

The scattering properties of a sample can affect the production of singlet oxygen [30] due to altered light fluence in the sample. Intralipid emulsion is commonly used as a light-scattering medium [31]. Here, 20% Intralipid (I141, Sigma Aldrich, St. Louis, MO, USA) diluted to 0.1–1% (after accounting for the 20% concentration of the stock commercial material) was added to a solution of 10 µg/g PpIX in 100% pure ethanol or acetone.

## 3. Results and Discussion

**Figure 4:**
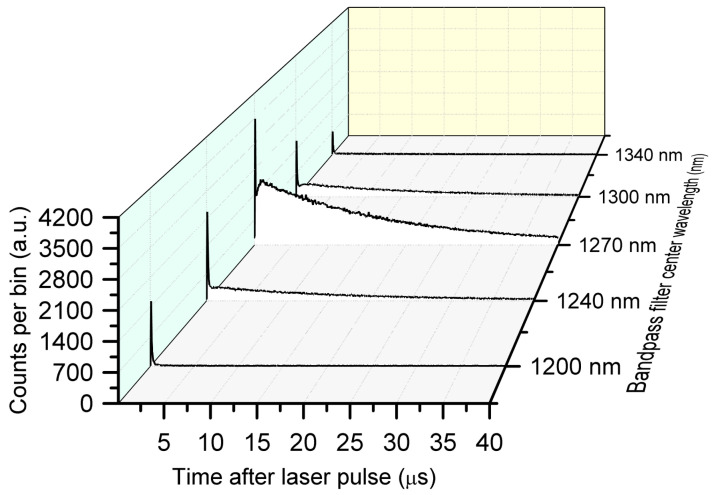
TCSPC histograms for 10 µg/g PpIX in ethanol with 10 min acquisition time using discrete band-pass filters showing the 1270 nm ^1^O_2_ luminescence peak and signal decay.

Figure 4 shows the results of measuring the singlet oxygen luminescence of 10 µg/g PpIX in ethanol using the multiple band-pass filters.

**Figure 5:**
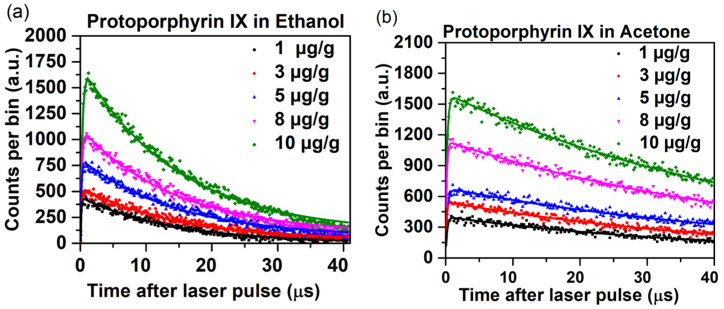
TSOLD single-photon counting time curves (measured dots, fitted lines) at 1270 nm for 1–10 µg/g PpIX in (**a**) ethanol and (**b**) acetone. 10 min integration.

Figure 5 shows the 1270 nm luminescence over time data for PpIX at different concentrations in ethanol and acetone. The derived ^1^O_2_ and photosensitizer triplet-state lifetimes are summarized in Table 1.

As expected, increasing the PpIX concentration had a minimal effect on the ^1^O_2_ lifetime in both solvents, but markedly increased the triplet-state lifetimes. The concentration-dependent triplet-state lifetime trend is consistent with previously reported data by Gemmell et al. [19] and is due to the high dose of PpIX.

**Figure 6:**
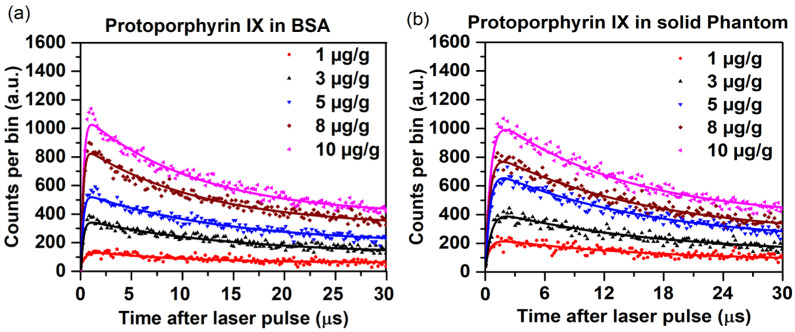
TSOLD single-photon counting time curves (measured dots, fitted lines) at 1270 for 1–10 µg/g PpIX at different concentrations in (**a**) BSA and (**b**) solid agarose. 15 min integration time.

The corresponding time histograms for PpIX in BSA and the solid phantom with different mass concentrations are shown in Figure 6.

From Figure 6, it is evident that the optimized SPAD efficiency and setup allow time-resolved detection of PpIX in biological-type media, despite the very weak signal. The total time-integrated counts increased 5–6-fold as the PpIX concentration increased from 1 to 10 µg/g. The corresponding ^1^O_2_ and triplet-state lifetimes are shown in Table 2.

The concentration of PpIX in agarose solid phantom directly impacts the ^1^O_2_ and triplet-state lifetimes. As the concentration of PpIX increases, excitonic interactions resulting from PPIX stacking and self-absorption become more pronounced, which causes a reduced triplet-state lifetime due to increased quenching and energy dissipation among closely packed PpIX molecules [32,33,34]. Meanwhile, the ^1^O_2_ lifetime increases with concentration in both BSA and agarose due to more efficient energy transfer to oxygen and potentially reduced non-radiative decay in the denser PpIX environment [35]. The photophysical properties and hydrophobicity of the surrounding medium are influenced by the specific interactions of PpIX with its microenvironment, such as protein binding in BSA or the gel matrix in agarose phantoms [36]. These effects are absent in the non-biological solvents. Also, the increase in viscosity of agar can impede the diffusion of singlet oxygen, consequently impacting its longevity and transport mechanisms. The reported studies showed that an increase in viscosity results in a decreased diffusion coefficient for singlet oxygen, thereby reducing diffusion distances and modifying its reactivity [37].

**Figure 7:**
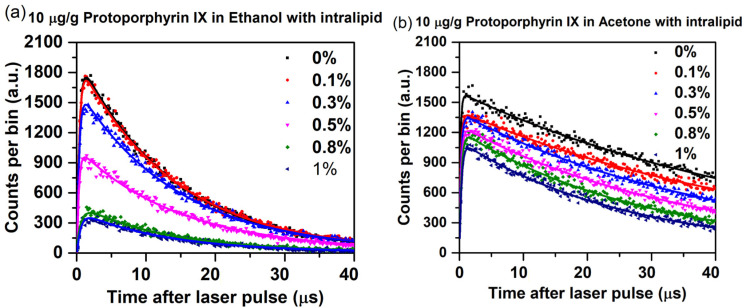
Measured and fitted luminescence plots for 10 µg/g PpIX in (**a**) ethanol and (**b**) acetone at Intralipid concentrations up to 1% using a 10 min acquisition time.

The time curves and fits by using Equation 1 for 10 µg/g PpIX in ethanol and acetone with different concentrations of added Intralipid are shown in Figure 7. The ^1^O_2_ luminescence counts decreased with increasing Intralipid concentration by ~5-fold and ~1.5-fold in ethanol and acetone, respectively. Qualitatively, this can be attributed to the increased scattering of the medium the excitation, and collected light reduces ^1^O_2_ luminescence detection [17,38]. The corresponding ^1^O_2_ and PpIX triplet-state lifetimes are shown in Table 3.

The addition of Intralipid has only a minor impact on the lifetime of singlet oxygen, except for some concentration-dependent reduction with acetone. Also, the PpIX binding to lipoproteins can alter the lifetime of singlet oxygen, which may occur due to the quenching of singlet oxygen by lipoprotein [39,40]. The PpIX triplet-state lifetime is significantly higher in ethanol in the presence of Intralipid and increases markedly with Intralipid concentration, while this effect is much less pronounced with acetone. Increased scattering can result in a prolonged residence time of the triplet state in the excited state, leading to diminished energy transfer efficiency and an extension of the triplet lifetime [41]. Due to different solubility properties of Intralipid in ethanol and acetone, variations in triplet-state lifetime are observed [42]. The residence time of the triplet state in the excited state can range from 100 ns to 10 s, contingent upon intrinsic factors, such as molecular photophysics, as well as extrinsic factors including environmental conditions and quenching agents [43]. Although the scattering properties of the solution do not exert a direct influence on the lifetime, they may affect measurements indirectly by altering signal detection within the scattering medium. Furthermore, variations in oxygen concentration can have a pronounced effect on triplet decay, as this can lead to quenching of the triplet state of protoporphyrin IX (PPIX), resulting in a reduced lifetime through chemical or collisional interactions.

The illumination laser diode (520 nm) wavelength was selected corresponding to the secondary absorption region of PpIX, which allows more effective excitation as well as minimizing background autofluorescence as compared to blue light. Also, the corresponding absorption coefficients of PpIX are higher at 520 nm than red wavelength which makes a better choice for studies such as optimizing singlet oxygen generation and detection under specific experimental conditions.

Finally, we note that, as with other reactive oxygen species, antioxidants can quench ^1^O_2_. This is used, for example, as a protective mechanism against oxidative damage in plants [44]. In mammalian cells, Soares et al. [45] have reported that the local oxidative stress produced by ^1^O_2_ during PDT can be mitigated through three main antioxidant mechanisms, namely superoxide dismutase, catalase and glutathione, and that the magnitude of this protective effect is cell type-dependent because of different endogenous levels of these reactants. An important translational issue is whether the “therapeutic window” for PDT damage to tumor versus normal tissues is impacted by their having different concentrations of antioxidants or could be enhanced by the tissue-specific administration of antioxidants. Direct ^1^O_2_ luminescence, as presented here, provides an excellent tool for studying these antioxidant effects directly and quantitatively without the potentially confounding use of secondary fluorescent reporters such as ^1^O_2_ sensor green.

## 4. Conclusions

The above results showed that the singlet oxygen (^1^O_2_) lifetime in PpIX photosensitizer is solvent- and microenvironment-dependent, ranging from ~14, ~49, ~29 and ~29 µs in ethanol, acetone, BSA and solid agarose, respectively. Acetone generally exhibits fewer non-radiative decay pathways for singlet oxygen in comparison to ethanol and biological media. This is attributed to the absence of hydrogen bonding, thereby leading to an extended lifetime [46,47,48]. The longer lifetime of ^1^O_2_ in BSA and agarose phantom, compared to ethanol, can be attributed to a combination of factors, including quenching interactions facilitated by protective protein interactions, viscosity, restricted diffusion due to the presence of hydrophilic and hydrophobic grooves, and physical properties (air-filled porosity, bulk density, shrinkage, etc.) of the biological media [49,50,51]. The singlet oxygen is highly reactive and short-lived; therefore, a small variation in ^1^O_2_ lifetime alters its reactivity and diffusivity. Esben Skovsen et al. (2005) demonstrated that singlet oxygen can have a surprisingly long lifetime within cells, allowing it to diffuse over considerable distances [52]. Also, a shorter lifetime of triplet-state photosensitizers such as 0.16 µs has been reported in BSA compared to agarose or ethanol due to quenching interactions with the protein and the complex microenvironment within BSA [51,53].

In the context of Intralipid studies, it has been observed that the binding of PpIX to lipoproteins has the potential to modify the lifetime of singlet oxygen. This modification arises from the possible quenching of singlet oxygen by lipoproteins [40]. The phenomenon of increased scattering can lead to a prolonged residence time of the triplet state in the excited state. Consequently, this can result in diminished energy transfer efficiency and an extension of the triplet lifetime [41]. Overall, the effects on the measurement are altered due to light scattering and possible binding/interactions of the PpIX with the Intralipid lipoproteins.

The TSOLD system used in this study is a significant advance over earlier instruments [16,17], featuring a nanosecond pulsed diode laser with a tunable pulse width and pulse rate capabilities. Notably, this system is considerably more compact and lower-cost than its predecessors [19,29]. The system’s performance was confirmed through the detection of the ^1^O_2_ signal from PpIX in ethanol, where the 1270 nm counts were significantly higher relative to the background than published values [19]. In the next stage of system engineering, the bifurcated fiber bundle coupled with an ns pulsed laser will further reduce the footprint of the system, further improving its practicality, especially for in vivo and clinical use.

In summary, the measurement of the PpIX concentration dependence of the luminescence generation in different solvents and biological media demonstrated that the ^1^O_2_ generation and lifetime are affected by changes in microenvironmental factors. Optical scattering in biological tissues also affects the spatial distribution of excitation light, and thereby of the ^1^O_2_ generation, as well as the near-infrared luminescence [54,55]. Also, quenching and diffusion limitations in biological media further reduce the ^1^O_2_ lifetime [54]. Hence, these multiple factors need to be taken into account during ^1^O_2_ luminescence measurements for PDT dosimetry, especially where absolute singlet oxygen concentrations are used to predict or correlate with the biological outcomes of the treatment. In addition, the singlet oxygen lifetime directly affects the effective diffusion range at cellular/subcellular scales [51], and previous studies have shown that, because of the very short ^1^O_2_ lifetime, the localization of the photosensitizer in different organelles has a marked impact on the cytotoxicity even for the same concentration of singlet oxygen [56].

## Figures and Tables

**Table 1:** Singlet oxygen and photosensitizer triplet-state PpIX lifetimes in ethanol and acetone.

PpIX Concentration (µg/g)	PpIX in Ethanol		PpIX in Acetone	
	Singlet Oxygen Lifetime (µs)	PpIX Triplet-State Lifetime (µs)	Singlet Oxygen Lifetime (µs)	PpIX Triplet-State Lifetime (µs)
1	13.8	0.10	47.9	0.16
3	14.4	0.13	48.2	0.21
5	14.6	0.14	48.3	0.27
8	14.4	0.18	48.4	0.17
10	14.6	0.22	48.6	0.25

**Table 2:** Singlet oxygen and photosensitizer triplet-state PpIX lifetimes in BSA and solid agarose, as per Table 1.

PpIX Concentration (µg/g)	PpIX in BSA		PpIX in Agarose-Based Solid Phantoms	
	Singlet Oxygen Lifetime (µs)	PpIX Triplet-State Lifetime (µs)	Singlet Oxygen Lifetime (µs)	PpIX Triplet-State Lifetime (µs)
1	18.9	0.51	21.1	0.54
3	23.0	0.20	25.4	0.44
5	26.9	0.19	27.5	0.38
8	28.4	0.17	28.8	0.36
10	28.5	0.16	28.9	0.34

**Table 3:** Singlet oxygen and PpIX triplet-state lifetimes for 10 µg/g PpIX in ethanol and acetone with varying Intralipid concentration.

Intralipid Concentration (%)	PpIX in Ethanol		PpIX in Acetone	
	Singlet Oxygen Lifetime (µs)	PpIX Triplet-State Lifetime (µs)	Singlet Oxygen Lifetime (µs)	PpIX Triplet-State Lifetime (µs)
0	14.6	0.30	48.6	0.18
0.1	14.4	0.32	48.0	0.24
0.3	14.2	0.34	45.9	0.25
0.5	13.8	0.36	42.1	0.28
0.8	13.3	0.51	40.4	0.31
1.0	12.7	0.55	38.7	0.33

## Data Availability

The raw data supporting the conclusions of this article will be made available by the authors on request.
